# Viral Zoonoses of National Importance in Ghana: Advancements and Opportunities for Enhancing Capacities for Early Detection and Response

**DOI:** 10.1155/2021/8938530

**Published:** 2021-01-15

**Authors:** Richard D. Suu-Ire, Evangeline Obodai, J. H. Kofi Bonney, Samuel O. Bel-Nono, William Ampofo, Terra R. Kelly

**Affiliations:** ^1^School of Veterinary Medicine, University of Ghana, Legon, Accra, Ghana; ^2^Noguchi Memorial Institute for Medical Research, University of Ghana, Off Akilagpa Sawyerr Road, Legon, Accra, Ghana; ^3^Military Veterinarian (Rtd.), P.O. Box CT2585, Accra, Ghana; ^4^One Health Institute, University of California, Davis, 1089 Veterinary Medicine Drive, CA 95616, USA

## Abstract

Zoonotic diseases have devastating impacts on human and animal health, livelihoods, and economies. Addressing the complex web of interrelated factors leading to zoonotic disease emergence and spread requires a transdisciplinary, cross-sectoral approach, One Health. The One Health approach, which considers the linkages between the health of people, animals, and their shared environment, presents opportunities to reduce these impacts through a more holistic coordinated strategy to understanding and mitigating disease risks. Understanding the linkages between animal, human, and environmental health risks and outcomes is critical for developing early detection systems and risk reduction strategies to address known and novel zoonotic disease threats. Nearly 70 countries across the world, including Ghana, have signed on to the Global Health Security Agenda (GHSA), which is facilitating multisectoral approaches to strengthen country capacities in the prevention and early detection of and respond to infectious disease threats. Currently, Ghana has not yet formalized a national One Health policy. The lack of a clearly defined multisectoral platform and limited collaboration among key Ghanaian Ministries, Departments, and Agencies has impacted the country's ability to effectively mitigate and respond to emerging and reemerging zoonoses. Many of these emerging zoonoses are caused by viruses, which, because of their diversity and evolutionary properties, are perceived to pose the greatest threat to global health security. Here, we review viral zoonoses of national importance and priority in Ghana, highlight recent advancements in One Health capacities, and discuss opportunities for implementing One Health approaches to mitigate zoonotic disease threats.

## 1. Introduction

Until recently, coordination among the human, animal, and environmental health sectors to address common disease threats has been limited [1]. This is of concern in many countries, where spillover of pathogens, especially viruses, from animals into humans present major challenges for health security [2]. Emerging infectious diseases (EIDs), the majority of which are zoonotic and originate in wildlife, pose substantial threats globally as evidenced by the devastating Ebola virus disease (EVD) outbreak in West Africa and the ongoing COVID-19 pandemic.

Although humans have coexisted with wildlife for thousands of years, recent population growth and the resultant anthropogenic pressures have intensified the interactions between people and animals, and consequently, human exposure to sylvatic disease cycles and the risk of pathogen spillover [3–6]. Anthropogenic factors driving these increased interactions include farming, extractive industries, hunting and trade of wild animals, and access to anthropogenic food sources among protected wildlife living in human-dominated landscapes [7–10]. Expansion of livestock production, especially in proximity to wildlife habitat, has also facilitated pathogen sharing in which livestock have served as amplifying hosts for zoonoses in people [11, 12]. Travel and human migration are also increasingly contributing to the emergence and spread of zoonotic diseases [13–15].

Preventing pathogen spillover, reducing cross-species transmission and spread, and minimizing the health and economic impacts of zoonotic disease threats require a paradigm shift in our approach to addressing health challenges at the animal-human-environment interface [16]. One Health, which recognizes the intrinsic linkages between the health of humans, animals, and their shared environment, is a cross-sectoral, transdisciplinary approach that provides opportunities for professionals to work collaboratively to solve complex health problems [16]. Transdisciplinary collaborations are important for improving our understanding of pathogen dynamics at the interface between animals, people, and the environment and can provide opportunities for prevention or early detection and control of disease threats before they lead to widespread outbreaks.

Nearly 70 countries across the world have signed on to the Global Health Security Agenda (GHSA), which is promoting One Health approaches to strengthen capacities in the prevention and early detection of and response to human and animal infectious disease threats [17]. Ghana became a member country of the GHSA in 2016. Since that time, stakeholders among the human, animal, and environmental health agencies and other relevant sectors in Ghana have come together to identify gaps in Ghana's health security system and strengthen the country's preparedness for health threats, including zoonoses. As part of this effort, Ghana conducted a national One Health Zoonotic Disease Prioritization workshop where representatives from the human, animal, and environmental health sectors and other partners prioritized zoonotic diseases of greatest concern in the country. Rabies, zoonotic avian influenza, and viral hemorrhagic fevers (Ebola virus disease, Lassa fever disease, yellow fever, and dengue hemorrhagic fever) were among the priority zoonoses. Here, we review viral zoonoses of national importance and priority in Ghana, present recent advancements in capacities for early detection and response to zoonotic disease threats, and highlight opportunities for implementing One Health approaches to mitigate these threats.

## 2. Methodology

### 2.1. Literature

We searched the peer-reviewed literature using online databases, including Web of Science Core, BIOSIS Previews, PubMed, and CAB Direct. Gray literature searches were also conducted using the University of Ghana document repository, which includes students' theses and dissertations; the World Health Organization web page; the Food and Agriculture Organization of the United Nations document repository; the World Organization for Animal Health (OIE) database; Google Scholar; and Government of Ghana disease reports. The keywords used in the search included different combinations of “viral,” “zoonoses,” “zoonotic,” “pathogens,” “diseases,” and “Ghana” with the Boolean operators “OR” and “AND.” Literature extending back to the earliest date for each database through 2019 was included. The titles and abstracts were screened for relevance to viral zoonoses in Ghana. Publications were excluded if they were not relevant, focused on viral zoonoses in laboratory animal settings, or were opinion papers without original or cited reports of zoonotic viruses. Reference lists from publications were also used to find relevant sources. Information extracted from each source included the virology, ecology and epidemiology, and public health impacts of the viruses in Ghana.

## 3. Results

Seventy-nine (79) publications were included in the review. Fifty-one (51) of these articles reported viral zoonoses in animals and 13 reported detections of zoonotic viruses in humans. The remaining 15 were general articles on viral zoonoses. Forty-four (44) of the publications discussed evidence of recently emerging viral zoonoses or viruses with unknown zoonotic potential but belonging to families with high-consequence zoonotic viruses, including avian influenza viruses (HPAI H5N1), coronaviruses (*Betacoronavirus*), and paramyxoviruses (Achimota virus 1 and Achimota virus 2 and *henipaviruses*).

### 3.1. Priority Viral Zoonoses in Ghana

#### 3.1.1. Rabies

Rabies virus, a *Lyssavirus* in the *Rhabdoviridae* family, is among the six *Lyssavirus* species reported to date in African mammals. Classical rabies is an underreported and neglected disease in low-income countries where capacities for rabies surveillance and diagnosis are generally more limited [18]. It is endemic across Africa where the domestic dog (*Canis familiaris*) serves as the principal reservoir and is responsible for nearly all (∼99%) reported human rabies cases [19]. Transmission of the virus is perpetuated through large numbers of stray dogs and unvaccinated pets. In Ghana, only 10% of dogs are vaccinated for rabies, and each year, there are approximately 100 human cases [20]. While both rabies and animal bites are notifiable events in Ghana, limited surveillance leads to gaps in detection and reporting [21], resulting in an underestimation of the burden of this disease.

In Ghana, the epidemiology of rabies virus is poorly understood mainly due to a lack of characterization of the circulating strains [22]. Phylogenetic analyses of viral sequences generated from animal rabies cases in Ghana from 2007–2009 confirmed the circulation of two sub-Saharan rabies virus lineages (Africa 1 and 2) [22] building on previous studies documenting only a single lineage (Africa 2) in West Africa. This finding in combination with phylogeographic analyses providing evidence of multiple introductions of viruses from other West African countries suggests a complex epidemiology and the need for a regional approach to rabies control [22].

Ghana has developed a national strategy for rabies prevention and control [23] and is participating in the global challenge to eliminate dog-transmitted human rabies by 2030. Initial control efforts have resulted in several positive outcomes, including the engagement of key One Health stakeholders, official sharing of rabies data between the veterinary and human health sectors, and cross-sectoral coordination on rabies prevention and control measures [24].

#### 3.1.2. Influenza A

Avian influenza viruses, which belong to the *Orthomyxoviridae* family of RNA viruses, infect a wide range of avian and mammalian hosts, with wild birds serving as the primary reservoir [25–27]. Relatively little is known about the emergence and circulation of avian influenza viruses among poultry populations in Africa. Outbreaks of highly pathogenic avian influenza H5N1 (HPAI H5N1) were first reported in African chickens in 2006, with initial reports from Nigeria [28]. The virus then spread to several countries, leading to large economic losses [28, 29]. In Ghana, the first case of HPAI H5N1 was detected in 2007 [30]. Since then, numerous outbreaks of influenza A have been detected, including an outbreak caused by a new subtype (H9N2) in commercial poultry in 2018 [31]. These outbreaks have had devastating impacts on the poultry industry in Ghana with more than 40,000 birds culled and USD $160,000 distributed to farmers in 2007 as compensation for losses due to HPAI H5N1 [30]. Migratory birds were hypothesized as the source for introduction and spread of these viruses in Ghana. However, influenza A viruses have not been detected in wild birds sampled during active disease surveillance efforts [32]. The borders are porous in West Africa, possibly facilitating the spread of the virus via the live poultry trade across the subregion.

In response to the HPAI H5N1 threat in humans, the Ghana Health Service (GHS) and the National Influenza Centre established human sentinel surveillance sites in regions where the HPAI H5N1 virus had been detected in poultry. Human surveillance for influenza viruses was later scaled-up under the Integrated Disease Surveillance and Response (IDSR) strategy to cover all regions during the 2009 influenza A H1N1 pandemic. From 2009 to 2010, there was a large increase in the number of influenza cases detected among children in Ghana, largely due to influenza A(H1N1) pdm09. This strain accounted for 86% of all type A influenza cases identified in 2010. The higher incidence may have been the result of heightened awareness and increased testing of children following press coverage of the pandemic [33]. In 2011, there was a shift in the prevalence with 35% (123/348) of confirmed influenza cases identified as Influenza A (H1N1) pdm09, 41 (11.8%) were Influenza A (H3N2), and 184 (53%) were Influenza B [34]. In 2017, an outbreak of influenza caused by the H1N1 pdm09 strain led to ninety-six infections and four deaths among students at a senior high school in Ghana illustrating the seriousness of seasonal influenza as a public health problem [35].

#### 3.1.3. Viral Hemorrhagic Fevers

Viral hemorrhagic fevers (VHFs) are caused by several families of viruses: *Arenaviridae*, *Bunyaviridae*, *Filoviridae*, *Flaviviridae*, and *Rhabdoviridae* [36, 37]. The majority of hemorrhagic fever viruses are zoonotic primarily spilling over into human hosts through the bite of an infected arthropod (tick and mosquito) or contact with a wild animal host [38].

Lassa fever virus (LASV), a single-stranded RNA virus of the *Arenaviridae* family, is endemic to West Africa. It is transmitted through contact with excretions from infected *Mastomys natalensis* and other rodent species [39]. The disease has been reported in Nigeria, Sierra Leone, Guinea, Liberia, Benin, Ghana, and Mali [39]. In Ghana, the first laboratory-confirmed cases of Lassa fever were detected in 2011 in the Ashanti Region [40] where the victims may have been exposed through contact with rodents during farming, hunting, and surface mining practices [40]. The disease was also reported in two Ghanaian soldiers on a United Nations peacekeeping mission in Liberia [14] and in a patient in Germany who had a recent history of travel to Ghana, Cote D'Ivoire, and Burkina Faso [41]. While infrequently detected in Ghana, the virus is common in other West African countries, with an estimated 300,000–500,000 cases and 5,000 deaths annually [42].

Yellow fever (YF), an acute viral hemorrhagic disease caused by a *Flavivirus*, is transmitted to people primarily through mosquito bites. Non-human primates serve as the primary reservoir, and mosquitoes acquire and transmit the virus by feeding on infected humans or non-human primates (WHO 2020). Epidemiological data over the past two decades attest to the resurgence of YF outbreaks in sub-Saharan Africa, with more than 1 million cases between 1986 and 1990. In Ghana, YF is considered endemic [43]. The trend in YF outbreaks seems to have a 10-year cycle in endemic countries [43]. In Africa, children under the age of 15 years now account for 70–90% of the cases [43, 44]. Though there have been advances in immunization programs, there is a need for sustained high immunization coverages to prevent outbreaks [45]. The GHS reported 2,660 suspected yellow fever cases in 2019. Mortality can be high during outbreaks in Ghana with three outbreaks accounting for 400 deaths [43].

Dengue fever virus (DENV), another member of the *Flavivirus* genus, is endemic in 34 countries across Africa [46]. In 2016, the world experienced several DENV outbreaks, including an outbreak in Burkina Faso where 9,029 cases and 18 deaths were detected [47]. While DENV is not reported to cause large outbreaks in Ghana, it is poorly documented. However, DENV-2 and DENV-3 were detected in sera of four patients clinically suspected of Ebola virus [48] and serologic studies have documented evidence of previous exposure in children presenting with malaria (38). These findings bring to the fore the need for surveillance and monitoring to assess the heterogeneity of Ghana's dengue fever burden.

To date, there have not been any reports of filoviruses (e.g., Ebola and Marburg viruses) detected in animals and humans in Ghana. However, serologic studies have identified filovirus antibodies in five species of fruit bats in Ghana [49]. Additional research is needed to better understand the role of these bats as potential hosts of filoviruses and whether any undetected spillover events have occurred in the country.

Crimean-Congo Hemorrhagic Fever Virus (CCHFV), which is endemic in Africa, is caused by a tick-borne virus (*Nairovirus*) in the family *Bunyaviridae*. Although ixodid ticks serve as reservoirs and vectors for CCHFV, a variety of animals, such as cattle, sheep, goat, and camels, are considered amplifying hosts for the virus. CCHF is a considerable public health threat, which can have significant impacts on abattoir workers and healthcare personnel, especially in resource-poor countries [50]. In Ghana, exposure to CCHFV has been reported in slaughterhouse workers and the virus was detected in ticks from cattle [51]. The limited options available for treatment and management of infected persons underscore the need for surveillance and targeted prevention and control measures [52].

### 3.2. Novel and Emerging Viruses of National Concern

In Ghana, both known zoonotic viruses and novel viruses of unknown zoonotic potential (from taxonomic families that include high-consequence zoonotic viruses) have been detected in free-ranging wildlife. West Africa has been identified as a region vulnerable to zoonotic disease spillover [5, 53, 54] driven by a complex web of ecological, economic, social, political, and cultural factors, as evidenced by the devastating West Africa EVD outbreak. Mitigating potential risks through disease surveillance, early detection, and characterization presents opportunities for addressing pathogens early at the source before spilling over into domestic animals or people.

#### 3.2.1. *Henipavirus*

*Henipavirus* is a genus in the family *Paramyxoviridae*, which includes two highly pathogenic zoonotic viruses: Hendra virus and Nipah virus. Both viruses cause severe encephalitis in humans, with case fatality rates ranging from 40 to 100% [55–58]. Anti-henipavirus antibodies and RNA sequences of three novel viruses phylogenetically related to known henipaviruses have been detected in *Eidolon helvum* fruit bats in Ghana [59, 60]. In addition, nonneutralizing antibodies against henipaviruses have been detected in pigs [61]. Cases of human henipavirus infections have not been reported in Ghana, and the public health significance of these recently discovered novel viruses is unknown.

#### 3.2.2. Achimota Virus 1 and 2

Achimota virus 1 (AchPV1) and Achimota virus 2 (AchPV2) are novel rubulaviruses isolated from the urine of *E. helvum* fruit bats in Ghana [62]. The viruses are phylogenetically related to each other and to other Old World fruit bat-derived rubulaviruses. Antibodies to Achimota viruses have been reported in large urban populations of *E. helvum* in Ghana and Tanzania and in smaller island *E. helvum* populations in the Gulf of Guinea [62]. Longitudinal sampling of *E. helvum* indicates virus persistence within the population and suggests spread of AchPVs via horizontal transmission among bats [62]. There is serological evidence of human infection with AchPV2 in Ghana and Tanzania suggesting spillover; however, the viruses have not been associated with clinical disease. Challenge studies of AchPV1 and AchPV2 in laboratory animals revealed infections in ferrets and guinea pigs [63], providing additional evidence that the viruses can cross species barriers.

#### 3.2.3. Monkeypox Virus

Human monkeypox is a rare viral zoonosis endemic to central and western Africa. Monkeypox virus belongs to the genus *Orthopoxvirus* (OPXV), which is distributed throughout Africa. Reservoir hosts of monkeypox include primates and wild rodents [64]. An outbreak of human monkeypox in the U.S. in 2003 was associated with importation of three genera of rodents (*Cricetomys*, *Funisciurus,* and *Graphirus*) from Ghana [65–67]. Following the outbreak, rodents were captured in Ghana and antibodies were detected in *Cricetomys, Graphiurus, Funisciurus, and Heliosciurus* spp., and evidence of OPXV DNA was detected in tissues of *Cricetomys, Graphiurus, and Xerus* spp. [66] suggesting these species may be involved in the maintenance of monkeypox virus in Ghana and other African countries [65, 66]. Although no clinical infections with OPXV have been reported in humans in Ghana, antibodies against OPXV were detected in over 50% of individuals residing near the rodent trapping sites. These findings suggest limited or indirect exposure to infected rodents resulting in seroconversion, but asymptomatic infections [66].

#### 3.2.4. Potentially Zoonotic Coronaviruses (Human Betacoronavirus 2c EMC/2012-Related Viruses in Bats) and Kumasi Rhabdovirus

*Coronaviridae* is composed of four genera: alpha- and betacoronaviruses are found in mammals, whereas gamma- and deltacoronaviruses are primarily found in birds [68]. Currently, there are six recognized coronavirus types that can infect humans, of which two betacoronaviruses, namely, severe acute respiratory syndrome coronavirus (SARS-CoV) and Middle East respiratory syndrome coronavirus (MERS-CoV), are zoonotic and have caused highly pathogenic respiratory infections globally [69]. SARS-CoV first emerged in 2002-2003 in China and quickly spread across multiple countries [70]. Unlike SARS-CoV, transmission of MERS-CoV is geographically limited and reported cases have often stemmed from outbreaks within the Middle Eastern countries or recent travel to the region [71].

Recent studies in Ghana have identified novel clade 2c betacoronaviruses in bats which were phylogenetically related to the MERS-CoV EMC/2012 strain [72]. Subsequently, studies among domestic livestock (cattle, sheep, goats, and donkeys) did not show evidence of infection with human betacoronavirus 2c EMC/2012-related viruses and thus may not serve as hosts of clade 2c coronaviruses [73]. Whether cross-order host switches have occurred for this novel clade 2c remains unknown. However, the hunting of bats for bushmeat may facilitate host switching events that can cause human disease outbreaks.

Coronavirus disease 2019 (COVID-19), which is caused by SARS-CoV-2, has affected more than 70,000,000 people worldwide as of December 2020. In March 2020, Ghana joined other affected countries in confirming its first two COVID-19 cases [74]. A range of animals are susceptible to SARS-CoV-2 infection [75]. However, the source of SARS-CoV-2 remains unknown. In Ghana, more than 53,000 cases and 327 deaths have been reported as of December 14, 2020. The Government of Ghana has introduced several risk reduction measures, including border and school closures, mandatory mask wearing, and banning all public gathering to limit community transmission. The COVID-19 pandemic is a stark reminder of the on-going threat of emerging and reemerging zoonoses and the need for One Health surveillance, early detection and response, and a robust research agenda to improve our understanding of the factors influencing risk of spillover.

## 4. Discussion

### 4.1. Recent Advancements and Opportunities for Implementing One Health Approaches in Ghana for Early Detection and Control of Zoonotic Disease Threats

The significance of emerging zoonoses in terms of human health and economic burdens, exemplified by the ongoing SARS-CoV-2 pandemic, has brought these threats to the fore. The seriousness of these threats has called for a shift to a transdisciplinary, multisectoral One Health approach for the prevention and early detection of and response to emerging zoonotic pathogens.

Ghana has made tremendous progress towards the development of a National Action Plan for Health Security (NAPHS). In 2017, Ghana conducted a Joint External Evaluation (JEE) to identify critical gaps that exist in the health system preparedness and response capacity [76]. The evaluation documented several opportunities that Ghana can leverage to enhance capabilities for rapid detection and response to zoonotic disease threats, including multisectoral prioritization of zoonotic diseases that the human and animal sectors to address jointly, establishing surveillance systems for these priority zoonoses, increasing coordination and information sharing between the animal and human health surveillance units in the government to improve surveillance and outbreak response, strengthening of the national laboratory system to enhance the surveillance for zoonotic diseases, and One Health workforce development [77].

To address these priorities, Ghana used a multisectoral, One Health approach to prioritize endemic and emerging zoonotic diseases of major public health concern to be jointly addressed by the human, animal, and environmental health sectors [78]. Since then, the Ghana Health Service (GHS) and Veterinary Services Directorate (VSD) have made efforts to strengthen their national surveillance and laboratory systems for rapid detection and response to the prioritized zoonotic diseases. For example, VSD partnered with the Food and Agriculture Organization (FAO) to enhance country capacities on risk assessment, disease surveillance, and laboratory diagnostics for zoonotic pathogens. As part of this effort, the FAO developed the Event Mobile Application tool (EMA-i) to support early animal disease reporting at the national level [79]. The mobile application, which is an epidemiological tool applied at the field level, has enabled sharing of weekly human and animal disease surveillance data between the human and animal health sectors in Ghana. This event-based surveillance application provides a parallel system to complement the Integrated Disease Surveillance and Response framework utilized by the Ghana Health Service, which provides weekly reports on rabies, acute hemorrhagic fever syndrome, yellow fever, and severe acute respiratory syndrome among other prioritized diseases in humans.

The government also partnered with the USAID Emerging Threats Program PREDICT project to strengthen surveillance and laboratory diagnostic capabilities for both known and newly discovered viruses within virus groups of public health importance, such as filoviruses (including Ebola viruses), influenza viruses, paramyxoviruses, and coronaviruses. The virus discovery protocols are now included in the molecular diagnostic suite used to investigate undiagnosed illnesses in humans and animals across the country. In doing so, the project expanded the scope of national disease surveillance to include a focus on zoonotic viruses of public health significance and enhanced national capacities to detect novel viruses that may be the cause of undiagnosed illnesses in Ghana. VSD also joined forces with the Global Affairs Canada and the Canadian Food Inspection Agency to enhance institutional and human capacity to detect anthrax, avian influenza, and African swine fever [80]. The improved infrastructure and skills as a result of efforts such as those highlighted here contributed to the rapid ramp up and testing for SARS-CoV-2 with 15 COVID-19 accredited human and veterinary labs throughout the country [81].

There are several recent examples of efforts to advance One Health workforce capacity in Ghana. The Training Programs in Epidemiology and Public Health Interventions Network (TEPHINET) has played a critical role in One Health workforce development in the country. The Ghana Field Epidemiology and Laboratory Training Programme (GFELTP) provides in-service training in applied epidemiology and public health laboratory practice for scientists, physicians, veterinarians, and other health professionals. The program is based on the One Health concept in its approach to train multidisciplinary teams of frontline professionals [82]. The Afrique One consortium, supported through the Wellcome Trust, supports pre-service training and multidisciplinary research opportunities in zoonotic diseases for students in Ghana [83]. These efforts are supplying the workforce pipeline with the next generation of One Health leaders in Ghana.

Despite broad support for the One Health concept, the realities of budgeting, coordinating, and implementing cross-sectoral activities, has proven difficult in Ghana, as elsewhere [84]. Currently, as a result of the lack of a clearly defined multisectoral platform, collaboration on One Health issues across partners has been limited [85]. Moving forward, there is agreement that implementation of the Ghana National Action Plan for Health Security (2019 to 2023) to address capacity gaps should be based on a One Health approach and aligned with broader health systems strengthening plans rooted in a whole-of-government approach. The Ghana One Health Policy, which will be finalized by the end of the year, is to guide effective coordination, communication, and collaboration among MDAs to achieve the overall objectives of the One Health approach as a health security measure to improved response to zoonotic disease threats moving forward [85]. The policy will also guide the work of statutory entities, industry, nongovernmental, and civic organizations which are involved in, or seeking involvement in addressing human, animal, and environmental health at all levels [85].

## Data Availability

No data were used to support the findings of this study.
